# Inoculating against the spread of Islamophobic and radical-Islamist disinformation

**DOI:** 10.1186/s41235-021-00323-z

**Published:** 2021-08-19

**Authors:** Stephan Lewandowsky, Muhsin Yesilada

**Affiliations:** 1 School of Psychological Science, University of Bristol, 12A Priory Road, Bristol, BS8 1TU UK; 2 University of Western Australia, Perth, Australia

**Keywords:** Inoculation, Extremism, Islamophobia, Radical Islamism

## Abstract

We report the results of a preregistered study that tested the effectiveness of inoculating participants against Islamophobic and radical-Islamist disinformation. Participants in the experimental (inoculation) condition watched a video that explained common rhetorical markers of radical-Islamist and Islamophobic disinformation that had been identified in an analysis of YouTube content. The information was presented in a neutral context not involving Islam and focused on analysis of the misleading argumentation. The control group watched a video about an unrelated topic. Participants were then exposed to target videos with “gateway” content that constituted an entry point to potential Islamist or Islamophobic radicalization. Both videos contained numerous items of disinformation. Participants then answered a variety of questions such as how likely they were to share the video, their level of agreement, and their perceived accuracy of the video. Participants who had received the inoculation displayed less agreement with the video content, perceived the video as less reliable, and were less likely to share it in comparison with participants in the control group. The study provides support for the use of argument-based inoculation in combatting extremist messages.

**Supplementary Information:**

The online version contains supplementary material available at 10.1186/s41235-021-00323-z.

## Significance statement

Social media and other online platforms have contributed to Islamist and Islamophobic radicalization by increasingly sophisticated methods (Kruglanski et al., 2018). There is evidence that YouTube in particular offers rapid pathways towards radicalization, with Islamophobic content being offered to viewers in response to seemingly neutral search terms. The offline consequences of online radicalization on society are increasingly concerning. For example, Islamophobic incidents in the UK have increased by 26% from 2016 to 2017 (Atta et al., 2018), and between 2019 and 2020, 50% of religious hate crimes recorded by the British police were targeted against Muslims (Home Office, 2020). Interventions to protect the public against being misled by extremists are therefore urgently needed. One challenge facing any intervention is the dynamically varying nature of radicalizing content which makes timely deployment of rebuttals difficult. This study therefore built on existing psychological inoculation theory to provide people with protection against misleading rhetoric. Based on the analysis of the rhetorical techniques used by Islamophobic and Islamist videos on YouTube, we created a brief video that explained those misleading techniques to participants in a neutral context. When participants were subsequently exposed to Islamophobic and Islamist videos, they rated them as less reliable and agreed with them less than participants in a control condition who first watched an unrelated video. The results show that argument-based inoculation can make people more resilient to extremist disinformation. Crucially, the same inoculation message provided protection against both Islamist and Islamophobic radicalizing information, suggesting that argument-based inoculation can transfer broadly. The results have important practical implications because it provides an intervention against radicalization that can be developed and rolled out without requiring specific knowledge of radicalizing content.

Misinformation has taken center stage in current political discussion. Misinformation refers to information that is either false or inaccurate. When misinformation is spread intentionally, for example, in pursuit of a political agenda, we refer to it as disinformation. The potential dangers of misinformation and disinformation are well established. For example, misinformation about the link between vaccines and autism has led many people in the USA refusing to vaccinate their children, thereby putting them at risk (Smith et al., 2008).

At the same time, radicalization and extremism are also growing global concerns. In a mutually reinforcing cycle known as reciprocal radicalization (e.g. Abbas, 2012, 2020; Abbas & Awan, 2015; Lee & Knott, 2020), Islamophobia and radical-Islamist views have gained prominence, sometimes resulting in extreme violence. In 2011, a right-wing extremist murdered 77 young people in Norway whom he presumed to be traitors conspiring to turn Norway into an Islamic society. In 2016, a jihadist extremist murdered 86 people in Nice, France, in retaliation against nations fighting the “Islamic State” in Syria and Iraq. Disinformation and propaganda are at the core of radicalization (e.g. Baugut & Neumann, 2019; Johnson, 2018).

Islamophobic portrayals in right-wing media facilitate Islamophobia (Bleich et al., 2015). Mentions of Islam in the press are more negative than mentions of other religious groups (Jaspal & Cinnirella, 2010). Similarly, right-wing media outlets consistently associate Islam with issues such as forced marriage and terrorism (Moore et al., 2008). Violent incidents involving Muslim perpetrators are readily labeled as “terrorism”, whereas equivalent acts by White perpetrators are labeled differently (see, e.g. Dolliver & Kearns, 2019). This pattern of coverage may explain negative public attitudes towards Islam. For example, 41% of US adults believe that Islam encourages violence more than other faiths, and 35% of these individuals believed that there was widespread extremism amongst US Muslims (Pew Research Center, 2017). These public opinions are in contrast to research indicating that 95% of Muslims believe “extremism and violence are never justified” (Ahmed & George, 2017; Pew Research Center, 2017).

The recognition of the importance of disinformation and propaganda in radicalization is not novel and applies equally to Islamist radicalization (e.g. Baugut & Neumann, 2019). Radical-Islamist groups use the internet to spread propaganda and seek recruits (Conway, 2017). For example, Islamic State claimed to be responsible for the 2017 mass shootings in Las Vegas; however, the FBI has since rejected these claims (Says, 2019). Likewise, in 2017 a doctored screenshot image of a sniper standing on a building in Colorado was released by Islamic State (Larson, 2018). It is likely that Islamic State used this false claim to spread fear and to radicalize individuals towards taking similar action. These activities affect search engines. For example, the originally benign religious term “Mujahideen” (which became common when describing soldiers from Afghanistan who fought against the British in the nineteenth Century; Farwell, 1985) returns radical-Islamist content on the second page of Google search results (Ahmed & George, 2017).

In response to Islamist misinformation, the US government has made repeated attempts to counter radicalization and jihadist-inspired terrorism by debunking misinformation and propaganda with a “Counter-Misinformation Team.” However, those efforts have not only been unsuccessful but may have been counterproductive (Aistrope, 2016). In part, this failure arose from a delegitimizing dynamic in the American discourse that undermined the intent to engage with a Muslim audience and instead caused further alienation (Aistrope, 2016). Although those specific errors might be avoidable by better design, in principle any persuasive effort or attempt to counter misinformation carries with it a risk of failure. There is evidence that the effectiveness of misinformation correction is mixed and often remains incomplete (Lewandowsky et al., 2012; Walter & Murphy, 2018). Another in-principle problem with addressing false information by specific rebuttals is the dynamically changing nature of disinformation. It takes seconds for information to go viral on social media and false information may spread further and faster than correct information (Vosoughi et al., 2018). Creating rebuttals, by contrast, takes time and care, and often fail to reach misinformed consumers (Guess et al., 2020).

These problems associated with countering misinformation may be avoided by interventions based on “inoculation theory” (Cook et al., 2017; Lewandowsky & van der Linden, 2021; van der Linden et al., 2017b). Inoculation equips individuals with the ability to critically assess and refute misinformation by revealing the flaws in misleading communications before exposure (Cook et al., 2017). Inoculation involves two components (van der Linden et al., 2017a). The first component is a reminder that politically motivated groups often distort or manipulate information in pursuit of their agenda. The second component explains the logical fallacies typically embedded in misinformation and provides a pre-emptive refutation (Roozenbeek & Linden, 2019).

Existing research has demonstrated that inoculation can protect the public against flawed contrarian argumentation about climate change and misinformation in general (Cook et al., 2017; Roozenbeek & van der Linden, 2019; van der Linden et al., 2017a, 2017b). In one study, inoculation was also found to create resistance to extremist propaganda (Braddock, 2019). Participants in that study were shown either an inoculation message or no-inoculation control message before reading left- or right-wing extremist propaganda. Inoculation reduced support for the extremist groups. The findings reported by Braddock provide an existence proof that inoculation may be a suitable tool to protect individuals against extremist messages. However, one limiting attribute of the study was that the inoculation messages were matched to the subsequent radicalizing material. That is, participants were inoculated against left-wing (right-wing) material by highlighting and rebutting specific left-wing (right-wing) claims. This leaves open the possibility that the inoculation observed by Braddock was narrow and constrained to the particular material being targeted.

Our study, by contrast, sought to inoculate participants against Islamist and Islamophobic radicalization using a common set of neutral, argument-based inoculation material. Our approach is anchored in two lines of relevant precedent: First, there is some evidence that the effects of inoculation can generalize across domains and specific instances. For example, Cook et al. (2017) presented participants with (a) a warning that the pervasive scientific consensus on climate change is often questioned for political reasons, and (b) an explanation that one such disinformation technique appeals to dissenting “fake experts” to feign a lack of scientific consensus. Cook and colleagues illustrated the “fake-expert” approach using the historical attempts of the tobacco industry to undermine the medical consensus about the health risks from smoking. Cook et al. (2017) found that exposing the fake-expert technique in one context (tobacco) inoculated individuals against the same technique in another context (climate change). This transfer is an important result because it suggests that inoculation can work even if it is focused on broader persuasion techniques rather than specific items of misinformation. Further support for the breadth of protection offered by inoculation was provided by Parker et al. (2012), who showed that if young people (college students) were successfully inoculated against one health-adverse behaviour (unprotected sex), the inoculation transferred to another risky behaviour (binge drinking).

Second, our approach relies on existing analyses of radicalization and violent extremism, which have identified rational cognitive processes, from knowledge acquisition to selective attention, that under certain circumstances can lead an individual to turn to violence in pursuit of their goals (e.g. Kruglanski et al., 2019). Contrary to popular views of radicalization and extremism as resulting from “brainwashing”, irrationality, or an assortment of psychological disorders, there is considerable evidence that the path to radicalization involves well-understood conventional cognitive processes (Kruglanski et al., 2018, 2019; Kruglanski et al., 2009; Moghaddam, 2005; van den Bos, 2020). The existing research on radicalization is thus at least broadly compatible with our assumption that argument-based inoculation—a quintessentially “rational” intervention—may increase people’s resilience to misleading rhetoric, and that it may do so irrespective of the specific polarity of the misleading rhetoric. If this were successful, it would demonstrate the success of a “broad spectrum vaccine” against potentially radicalizing disinformation. To our knowledge, inoculation has not been applied to Islamophobic and Radical-Islamist disinformation before.

We focused our intervention on YouTube. YouTube boasts over 2 billion users (YouTube, n.d.), making it the second most visited website worldwide. YouTube has also become home to political extremism of many colours, mainly on the extreme right (e.g. Kaiser & Rauchfleisch, 2020; Lewis, 2018; Rauchfleisch & Kaiser, 2020). At the heart of YouTube’s architecture is a recommender system that is designed to maximize viewing time on the platform (Covington et al., 2016). Each video on YouTube is accompanied by recommendations for further viewing in a sidebar. These recommendations are created by “intelligent” algorithms based on the user’s activity and the interconnectedness of videos. YouTube recommender algorithms have been repeatedly criticized for facilitating pathways to radicalizing content (Schmitt et al., 2018; Spinelli & Crovella, 2020). For example, users who viewed videos of Donald Trump during the 2016 presidential campaign were subsequently presented with videos featuring white supremacists and Holocaust denialists. After playing videos of Bernie Sanders, YouTube suggested videos relating to left-wing conspiracies, such as the claim that the US government was behind the September 11 attacks (Tufekci, 2018). A recent preregistered study of the YouTube recommender system confirmed that it was liable to promote and amplify conspiratorial content even in response to relatively innocuous search terms (Alfano et al., 2020).

A particularly troubling aspect of the algorithm is that it has difficulty differentiating between radical content and other messages. For example, radical content can appear in the recommender tab of far-right *counter*messages. That is, deradicalization messages on YouTube may be accompanied by recommendations to precisely the opposite (Schmitt et al., 2018). Moreover, an audit of pathways towards radicalization identified pathways between Alt-lite (a loosely defined right-wing group who see themselves separate from the far-right) videos and the Intellectual dark web (a group of political commentators who regard identity politics and political correctness as a danger to society). The analysis also uncovered pathways between Alt-right channels (white nationalist movements) and Intellectual dark web videos (Ribeiro et al., 2020). Our own analysis of YouTube (reported in the Additional file 1) likewise showed that Islamophobic content is strikingly easy to encounter on YouTube. For example, when the search string “Islam United Kingdom” is entered into YouTube’s home page (search done on 26 July 2021), one of the top 10 suggested videos features a far-right British personality who has referred to Islam as “repugnant” and has called immigrants to Britain “cockroaches” (Bridge Initiative Team, 2018). Citing her long-standing racist record, Twitter permanently banned her account in June 2020 (Robertson, 2020), but her YouTube channel remains active as of the date of this writing (July 2021). This search result is unlikely to be an isolated incident: A recent analysis of racist content on YouTube by Hokka (2021) concluded that “YouTube’s policies and practices as ideological choices contribute to the normalisation of racism on social media” (p. 142).

Overall, there is sufficient evidence to warrant concern about YouTube’s role in directing viewers to radical or extremist content. It is therefore particularly important to develop materials that can help viewers become resilient to such content and to resist the potential allure of radicalizing material.

## Method

In preparation for the current study, we analyzed Islamophobic and radical-Islamist videos on YouTube using the YTDT tool (Rieder, 2015) to understand the techniques by which extremists mislead. This analysis is reported in the Additional file 1. The present study used these rhetorical markers of misinformation to create inoculating tools that can protect vulnerable people against misinformation and potential Islamophobic and Islamist radicalization.

The Method and analysis plan were preregistered. The preregistration is available at https://osf.io/au9wh/.

### Participants

The number of required participants was calculated using the software G*power using *α* = 0.05, *f* = 0.15, resulting in a total required sample size of 580. Participants were recruited through the online platform Prolific and were paid £3.15 for the 30-min session. All participants resided in the U.K. at the time of participating. To compensate for drop-outs before completion, a total of 641 participants were recruited by Prolific, which yielded a final sample size of 591 participants (368 females, 218 males, 3 non-binary, and 1 withheld response).

The average age of participants was 35.50 (SD = 12.40). 4.2% of participants were Muslim, 33.2% were Christian, 36.4% were Atheist, 14.4% were Agnostic, 9% were Other, 1.4% were Hindu, 0.5% were Jewish, 0.3% were Sikh, and 0.7% were Buddhist.

### Design

The study used a 2 × 2 between-subjects design, with variables training (no intervention vs. inoculation) and misinformation (Islamophobic Misinformation vs. radical-Islamist misinformation). Participants were randomly allocated to one of the 4 groups (see Table 1 for the number of participants per group). Dependent variables were perceived accuracy of the target video, feelings of anger, likelihood to share the target video, extent of agreement and extent of support for the target video, and next-video preference (expressed by choosing another video from a “recommender system”).

**Table 1:** Number of participants per group

		Type of misinformation	
		Islamophobic	Islamist
Training condition	Inoculation	149	145
	Control	151	146

### Procedure

**Fig. 1:**
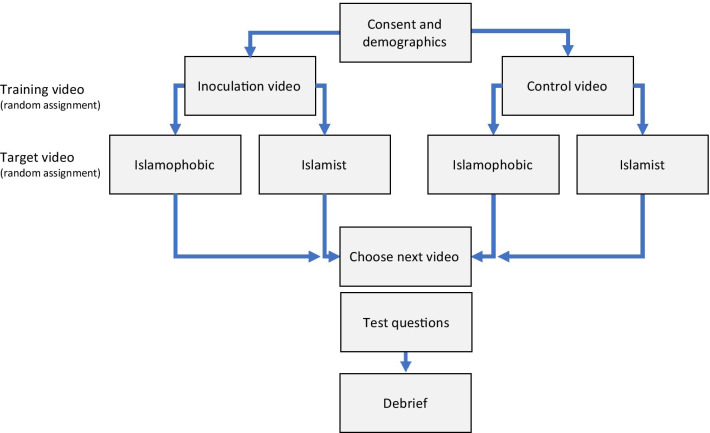
Overview of procedure

**Fig. 2:**
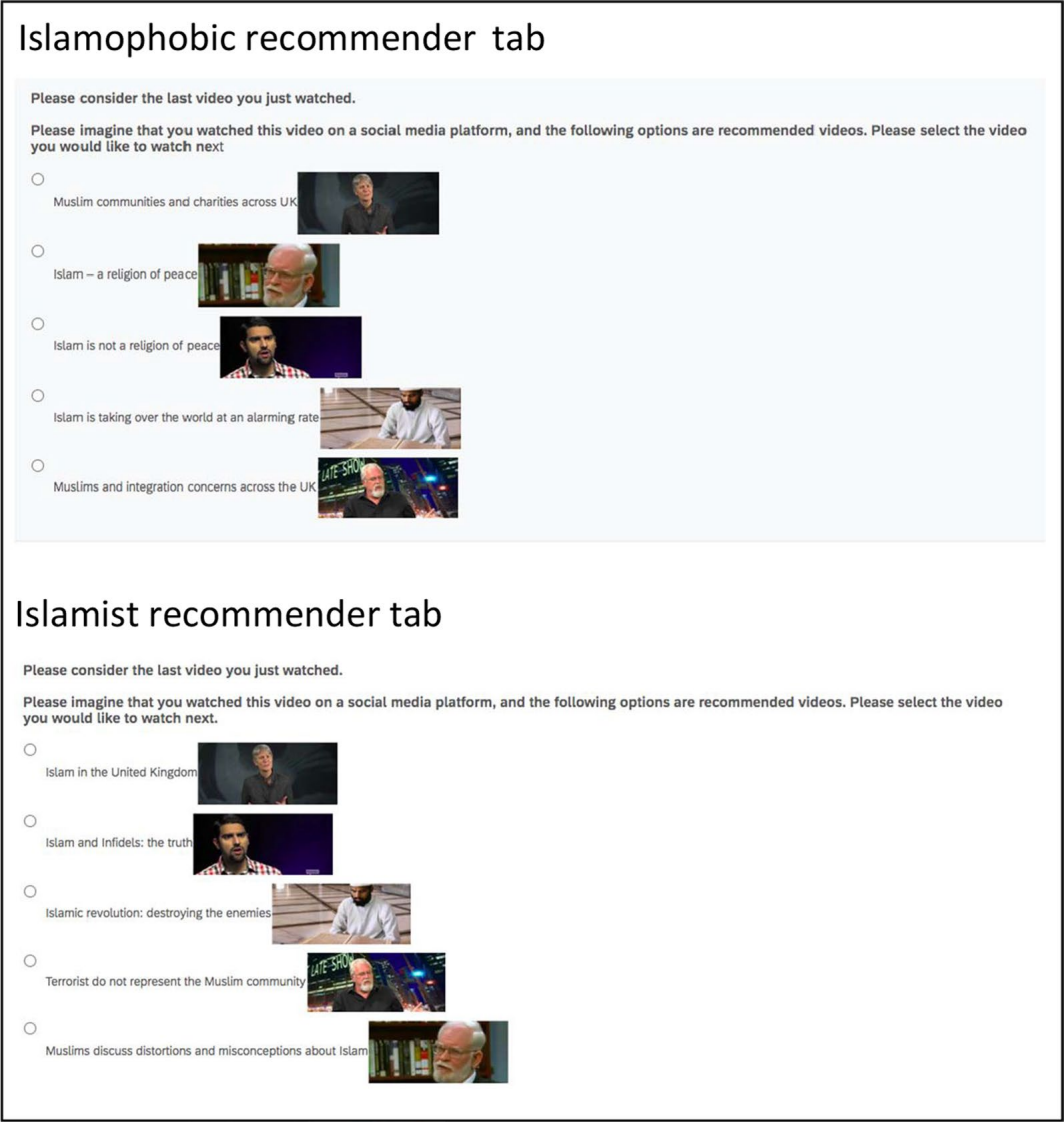
Screen snapshot of the recommender tabs for the two conditions

Figure 1 provides an overview of the procedure. Participants first answered demographic questions, including about their religious orientation. Participants then either watched the training material (inoculation condition; see below for details) or content about an unrelated issue (control condition). The control condition video taught participants about the use of bitcoin and the origin of money and was the same length as the inoculation video. Participants then watched the target video, which depending on random assignment either displayed content comprising a conduit to radical-Islamist content or Islamophobic content. All participants were then presented with a mock YouTube sidebar with a recommender tab of 5 videos (see Fig. 2) that, dependening on condition, displayed Islamophobic or radical-Islamist video titles. The titles and thumbnails were arranged on an ordinal scale of extremism, from benign content to extreme content. Participants were asked to select from the recommender tab what video they would like to watch next.

Following their next-video selection, participants responded to questions about the target video. All questions used a 5-point Likert scale, with the exception of agreement, which used a 6-point scale. The first question investigated participants’ likelihood of sharing the video via social media platforms (response options ranging from highly unlikely to highly likely). The second question inquired about the extent to which participants believed the video to be reliable (response options ranging from highly unreliable to highly reliable). The third question aimed to determine participants’ level of anger after watching the video (response options ranging from none at all to a great deal). The fourth question queried the extent to which participants agreed with the video (response options ranging from “I accepted all of the points made in the message” to “I argued against all of the points made in the message”). The fifth and final question aimed to determine participants’ level of support for the ideas presented in the video. Instead of a 5-point scale, this question used a slider from 0-100. The slider was positioned at 0 at the outset.

Participants were then asked to watch a debrief video and read a debrief sheet. The debrief video consisted of the inoculation video and a video explaining the push and pull factors involved in radicalization. Participants who did not watch the debrief video were sent an invitation to complete the study by watching the debrief video. Fifteen participants were sent an invitation to watch the debriefing video. Thirteen of these participants completed the debrief upon receiving the invitation. Data from participants who did not watch the debrief video during the experiment were included, irrespective of whether or not they subsequently followed the invitation.

## Materials

### Training video

The training video for the inoculation condition was designed to counter the prevailing misleading rhetorical techniques identified by our analysis of extremist YouTube videos (see Additional file 1: Section S1 for details). The analysis identified hasty generalizations, invoking emotion, and polarization as common markers of Islam-related misinformation. Polarization refers to the process of amplifying existing differences and tensions between different groups of people (Groenendyk, 2018). Hasty generalizations involve individuals jumping to conclusions based on incorrect induction and flawed statistical reasoning (Walton 2008, pp. 246–247). Invoking emotion is a persuasive technique in which individuals appeal to human emotions such as fear, anger, or empathy (e.g. Das et al., 2003). In the context of political persuasion, emotional language also tends to have a strong moral component (e.g. Brady et al., 2017), and moralization of content has been associated with the emergence of violence (Mooijman et al., 2018). The training video used a series of narrated animations to explain how each misinformation technique is used to mislead. The video did not mention Islam or any related issues but used hypothetical and generic examples from politics to explain the techniques. The video was 5 min long and is available at: https://vimeo.com/439769758/cf388de426.

### Target videos

The Islamophobic and radical-Islamist videos were designed by harvesting background video (“b-roll”) from Islam-related videos on YouTube. The scripts for the target videos used the three misleading techniques (hasty generalization, polarization and invoking emotion) in order. The emotional segments were suffused with moral language to mirror the role of moralization in actual political speech (e.g. Brady et al., 2017; Wang & Inbar, 2021). The scripts are available in the Additional file 1: Section S2. The videos themselves are available online but given the sensitivity of the information, they are not public. The links and passwords can be obtained from the authors upon request. To ensure comparability of scripts between the target videos, they were analysed using the Linguistic Inquiry and Word Count (LIWC) program (Pennebaker et al., 2015). The LIWC software analyzes text and counts the percentage of words that reflect different emotions, thinking styles, social concerns, and parts of speech. As shown in Table 2, the texts were similar in the word count for each rhetorical misinformation technique (hasty generalizations, invoking emotion, polarization) and in terms of the percentage of social words, positive words, and negative words used. The table also shows that the scripts used less negative emotion words and more positive emotion words compared to actual extremist content obtained from YouTube. The scripts also contain more negative and positive emotion words in comparison with neutral informational videos about Islam, also obtained from YouTube (links to these videos are provided in the table). A small pilot study on five participants was conducted to check if the scripts produced strong negative emotions. The scripts did not evoke emotional distress, anger, desire to harm others, or overall negative emotions.

**Table 2:** LIWC analysis of training videos and extremist videos on YouTube

Measure	Islamophobic script	Islamist script	YouTube	YouTube	YouTube	YouTube
			Islamo-phobic^a^	Islamist^b^	Neutral Islamo-phobic^c^	Neutral Islamist^d^
Hasty Generalizations word count	175.0	174.0				
Invoking Emotion word count	194.0	197.0				
Polarzation word count	169.0	168.0				
I words (I, me, my) (%)	0.2	0.2	1.0	2.9	0.7	0.0
Social Words (%)	8.2	7.8	14.6	15.6	7.8	9.1
Positive Emotions (%)	3.7	3.8	2.0	1.3	1.6	2.3
Negative Emotions (%)	1.7	1.7	5.3	2.6	0.0	1.4

^a^https://youtu.be/8T9JJi6kqrc

^b^removed from YouTube

^c^https://youtu.be/glAI5YMMw0Y

^d^https://youtu.be/sjJVO8GASmw

### Results

**Fig. 3:**
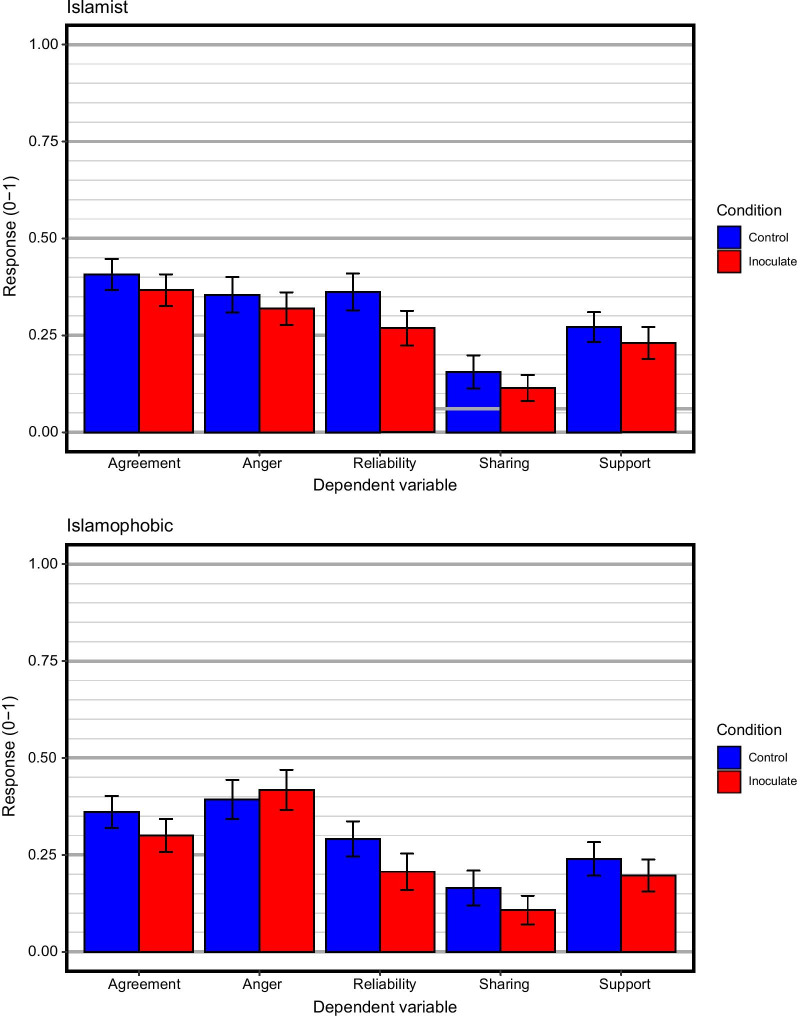
Summary of main dependent variables for all conditions. The top panel is for the Islamist target video and the bottom panel for the Islamophobic target video. All dependent variables are rescaled to the range 0 to 1 for commensurability. Error bars represent 95% confidence intervals

Figure 3 provides an overview of the results for the main dependent variables. The preregistered analysis plan (see https://osf.io/au9wh) prescribed independent 2 × 2 ANOVAs to test the effects of training condition and type of misinformation on the dependent variables (sharing likelihood, perceived reliability, anger, agreement, and support for the video). To put these ANOVAs into an overall context, we first performed a multivariate analysis (2 × 2 MANOVA) on all 5 dependent variables simultaneously. The MANOVA was not preregistered. The analysis yielded a significant main effect of training condition, *V* = 0.03, *F* (5, 528) = 3.37, *p* = .005. The main effect of type of misinformation fell just short of significance, *V* = 0.02, *F* (5, 528) = 2.19, *p* = .054. There was no interaction between the two experimental variables, *V* = 0.00, *F* (5, 528) = 0.23, *p* = .950. These omnibus effects were largely mirrored in the individual ANOVAs.

### Sharing likelihood

There was a significant main effect of training condition on sharing likelihood, *F* (1, 587) = 5.97, MSE = 0.96, *p* = *0.0*15, $\hat{\eta }_{2}^{G}$ = *0.0*10. Participants in the inoculation condition (*M* = 1.44, 95% CI: 1.34–1.54) were less likely to share the misinformation content than participants in the control condition (*M* = 1.64, 95% CI: 1.52–1.76). There was no main effect of type of misinformation, *F* (1, 587) = 0.00, MSE = 0.96, *p* = *0.9*54, $\hat{\eta }_{2}^{G}$ = *0.0*00, nor an interaction effect between training and misinformation, *F* (1, 587) = 0.14, MSE = 0.96, *p* = *0.7*08, $\hat{\eta }_{2}^{G}$ = *0.0*00.

### Perceived reliability

There were main effects of training condition, *F* (1, 586) = 14.11, MSE = 1.29, *p* < *0.0*01, $\hat{\eta }_{2}^{G}$ = *0.0*24, and type of misinformation, *F* (1, 586) = 8.10, MSE = 1.29, *p* = *0.0*05, $\hat{\eta }_{2}^{G}$ = *0.0*14, on perceived reliability. Participants in the inoculation condition perceived the misinformation content as less reliable than participants in the control group (*M* = 1.95, 95% CI: 1.82–2.08 vs. *M* = 2.30, 95% CI: 2.17–2.43). Participants who saw Islamophobic misinformation rated the content as less reliable than participants who saw radical-Islamist misinformation (*M* = 2.26, 95% CI: 2.13–2.39 vs. *M* = 2.00, 95% CI: 1.87–2.13). There was no interaction between the two experimental variables, *F* (1, 586) = 0.04, MSE = 1.29, *p* = *0.8*46, $\hat{\eta }_{2}^{G}$ = *0.0*00.

### Anger

There was a significant main effect of misinformation on participants’ feeling of anger, *F* (1, 586) = 8.02, MSE = 1.38, *p* = *0.0*05, $\hat{\eta }_{2}^{G}$ = *0.0*13. Participants who watched Islamophobic content reported greater feelings of anger than participants who watched radical-Islamist content (*M* = 2.35, 95% CI: 2.22–2.47 vs. *M* = 2.62, 95% CI: 2.48–2.76). There was no main effect of training condition, *F* (1, 586) = 0.08, MSE = 1.38, *p* = *0.7*77, $\hat{\eta }_{2}^{G}$ = *0.0*00, nor an.

interaction between the two experimental variables, *F* (1, 586) = 1.56, MSE = 1.38, *p* = *0.2*13, $\hat{\eta }_{2}^{G}$ = *0.0*03.

### Agreement

The main effects of training condition, *F* (1, 587) = 5.58, MSE = 1.62, *p* = *0.0*19, $\hat{\eta }_{2}^{G}$ = *0.0*09, and type of misinformation, *F* (1, 587) = 7.23, MSE = 1.62, *p* = *0.0*07, $\hat{\eta }_{2}^{G}$ = *0.0*12, were both significant. Participants who received inoculation agreed less with the misinformation content than participants in the control group (*M* = 2.67, 95% CI: 2.52–2.82 vs. *M* = 2.92, 95% CI: 2.77–3.06). Participants who watched the Islamophobic content agreed less with the points made in the video than participants who watched the radical-Islamist content (*M* = 2.93 vs. *M* = 2.66). There was no interaction between the two experimental variables, *F* (1, 587) = 0.23, MSE = 1.62, *p* = *0.6*31, $\hat{\eta }_{2}^{G}$ = *0.0*00.

### Support for the video

Unlike the other measures, the survey software recorded a notable number of missing responses for this measure. This likely reflected the fact that for this question, a slider was used, with the original position of the slider at zero. Thus, if a participant wanted to report zero support, they would have had to log a click on the slider and then move it back to zero. It is possible that some participants were not aware of this and proceeded to the next question without moving the slider, which was recorded as a missing response. There was a main effect of training condition, *F* (1, 534) = 3.49, MSE = 637.38, *p* = *0.0*62, $\hat{\eta }_{2}^{G}$ = *0.0*06. Participants in the inoculation condition indicated less support (*M* = 21.48, 95% CI: 18.44–24.51) than participants in the control group (*M* = 25.55, 95% CI: 22.52–28.57). There was no main effect of type of misinformation, *F* (1, 534) = 2.24, MSE = 637.38, *p* = *0.1*35, $\hat{\eta }_{2}^{G}$ = *0.0*04, nor an interaction between both variables, *F* (1, 534) = 0.00, MSE = 637.38, *p* = *0.9*59, $\hat{\eta }_{2}^{G}$ = 0.000, on participants’ level of support.

### Next-video response

We next analysed responses to the “recommender system” tab (Fig. 2). None of the effects were significant. There was no main effect of training condition, *F* (1, 587) = 0.16, MSE = 1.37, *p* = *0.6*86, $\hat{\eta }_{2}^{G}$ = *0.0*00, type of misinformation, *F* (1, 587) = 0.27, MSE = 1.37, *p* = *0.6*06, $\hat{\eta }_{2}^{G}$ = *0.0*00, and there was no interaction, *F* (1, 587) = 0.01, MSE = 1.37, *p* = *0.9*08, $\hat{\eta }_{2}^{G}$ = *0.0*00. One reason for this outcome might be that the videos offered in the recommender system tab were not perceived to have the intended clear ordinal relationship from lowest to highest extremity.

### Exploration of anger and agreement

**Fig. 4:**
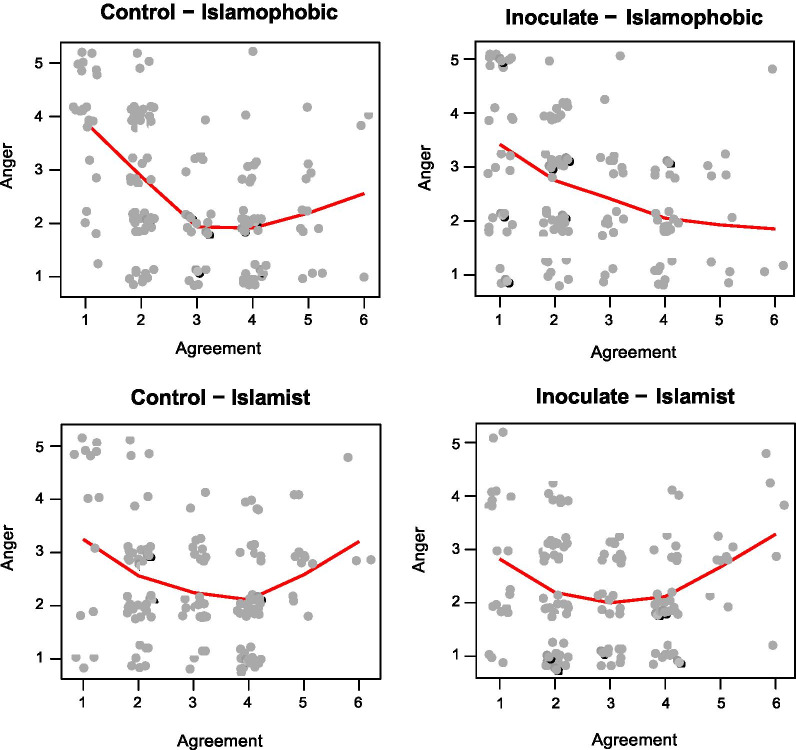
Relationship between anger and agreement with the video in the 4 conditions. All points are jittered to avoid overprinting. The red lines represent lowess smoothing

We conducted an additional exploratory analysis (not preregistered) that examined the association between self-reported anger and agreement with the target video. Figure 4 displays the results, broken down by condition. One might expect that low agreement with the video might be associated with greater anger. The figure shows that this association was indeed observed, to varying extents, in all conditions. Perhaps unexpectedly, anger was also greater when agreement was greatest, in 3 out of 4 of the conditions. A possible reason might be that anger is directed differently in the two situations: When agreement is low, anger might be directed at the content of the video, whereas if agreement is high, anger might be directed at the groups targeted *by* the video. This account is intriguing but speculative and we do not pursue it further.

## Discussion

### Limitations and relationship to previous results

Several limitations of the study must be recognized before we consider its implications. First, the study did not measure the duration of the inoculation effect. Previous research has found that inoculation treatments typically decay over a number of weeks (Banas & Rains, 2010; Niederdeppe et al., 2014; Zerback et al., 2020), much in line with the forgetting of rebuttals of misinformation (Swire et al., 2017). Recent results have pointed to the possibility that occasional “booster” doses can extend the retention of inoculation (Ivanov et al., 2018; Maertens et al., 2020). In the study by Maertens et al. (2020), the inoculation benefits of a misinformation game (which participants played for 15 min) were found to wear off after 2 months without further interventions. By contrast, the benefits retained intact for 3 months if the retention interval included repeated tests. Future studies should follow up on this intriguing suggestion that the effects of inoculation can be extended by brief “booster” episodes.

Second, the study did not investigate whether inoculation is superior to fact-checking or corrections. It is conceivable that a correction after exposure to the target video could have achieved a similar reduction in acceptance and sharing intentions and so on. The existing literature has not resolved this issue. On the one hand, Jolley and Douglas (2017) showed that inoculating people against anti-vaccination conspiracy theories was more effective than attempts to debunk a conspiracy theory after it had been presented. On the other hand, a recent large-scale study (*N* = 2,683) purported to show that debunking is more effective at improving truth discernment than inoculation-style “pre-bunking” (Brashier et al., 2021). However, that study regarded a simple “true” or false" flag after or before a contested claim as constituting “debunking” and “pre-bunking”, respectively. Simply identifying something as true or false does not constitute adequate inoculation which, by definition, requires a warning combined with a refutation of an anticipated argument. The study by Brashier and colleagues therefore cannot adjudicate the relative effectiveness of inoculation and debunking. Future research should compare the benefits of inoculation to other approaches of combatting misinformation. However, this question is mainly of theoretical interest because in a real-world context, rebuttals are more difficult to roll out at scale for the reasons noted at the outset—namely, the dynamically changing nature of disinformation and preferential uptake of false information over fact checks. Inoculation can overcome at least one of those problems because, as we show in our study, generic refutations of misleading arguments can succeed irrespective of the specific content that follows.

Third, one might be concerned that our results somehow reflected demand effects. Perhaps participants simply responded to our training video by endorsing whatever followed less on all dependent measures than they would have without the video. It is difficult to rule out this possibility completely, although we find it unlikely in light of other research that has shown that inoculation can boost individuals’ truth discernment ability (Basol et al., 2021). That is, in the study by Basol and colleagues, participants were tested with both true and false pieces of information, and their ability to differentiate between the two types of information (e.g. misinformation manipulativeness ratings minus real-news manipulativeness ratings) increased after inoculation compared to a control condition. This finding is difficult to explain as a demand effect because a blanket demand effect would have increased manipulativeness ratings for all types of material.

A fourth concern might centre on the fact that our sample was predominantly non-Muslim. One might therefore legitimately question the utility of presenting those participants with Islamist material, given that it likely has low a priori attractiveness to a non-Muslim audience. In response, we note that a perceived loss of personal significance is a core ingredient of radicalization (e.g. Kruglanski et al., 2018). The loss of personal significance, in turn, can make individuals susceptible to a broad range of radicalizing narratives, including some that may appear culturally distant. To illustrate, although estimates are imprecise, there likely are several hundred western citizens who converted to Islam and then fought for ISIS in Iraq (Schuurman et al., 2016). Inoculating non-Muslim audiences against Islamist disinformation may therefore address a small but known risk of radicalization.

Finally, critics might argue that our observed effect sizes were too small to have much practical impact. In response, we suggest that even small effects can have major repercussions if they are scaled up to the population at large. President Trump won the election in 2016 by a razor-thin margin in a few key states, equivalent in number to the capacity of a single football stadium, or .0009 of all votes cast (Meko et al., 2016). Clearly, even a very small intervention could have swung the outcome of the election. Other recent results support this contention. For example, Pennycook et al. (2020) showed that providing a simple accuracy reminder nearly tripled people’s truth discernment of headlines relating to COVID-19. Another real-life example involves small changes to the architecture of the WhatsApp platform. In India in 2018, false rumours about child kidnappers shared via WhatsApp’s unlimited forward facility were implicated in mob lynchings that led to 29 innocent people being killed (Dixit & Mac, 2018). In response, WhatsApp introduced several small changes to their app, including curtailing the number of recipients a message could be forwarded to (thereby preventing large cascades). These relatively small changes may have contributed to the subsequent cessation of lynch killings in India (de Freitas Melo et al., 2019), suggesting again that even small effects can scale up to considerable societal impact if they diminish the likelihood of viral cascades of misinformation online.

Although those limitations should not be ignored, we do not consider them sufficiently serious to prevent interpretation of our results. The principal contribution of our study is that it shows how generic argument-based inoculation in a neutral context can help combat extremist messages of opposing polarity. Although much is known about the effectiveness of inoculation against general misinformation (Cook et al., 2017; Roozenbeek & Linden, 2019; Roozenbeek et al., 2020; van der Linden et al., 2017a, 2017b), the evidence base relating to extremism is scarce. In addition to the study by Braddock (2019) mentioned earlier, we know of only one further study (Saleh et al., 2020). This recent study inoculated participants through an “active” manipulation, by inviting participants to play a game in which they pretended to be a recruiter for a fictitious terrorist group. This role-playing exercise was found to increase participants’ ability to detect manipulative messages. Although these results are promising, one limitation of the game approach (see also, Roozenbeek & Linden, 2019; Roozenbeek et al., 2020) is that the time involvement (15 min) is considerably higher than in other inoculation contexts, including our study (5 min). Duration of the treatment is a non-trivial problem because brevity is crucial for a large-scale rollout of an intervention.

### Theoretical implications

Our results also have implications for psychological theory. Perhaps most important is the finding that generic inoculation can extend into specific domains of diametrically opposed polarity. This result falls within a broader trend of findings that inoculation is not necessarily confined to a domain but may have “broad spectrum” appeal (for a review, see Lewandowsky and van der Linden, 2021). For example, as noted at the outset, Cook et al. (2017) showed that inoculation against fake-expert disinformation in one domain (misleading advertising by the tobacco industry) transferred to another domain (climate change).

The successful transfer of argumentation-based inoculation provides further impetus to scholarly analyses of flawed argumentation and how that is used to disinform. Inventories of flawed rhetoric have been compiled for populist politicians (Blassnig et al., 2019), anti-vaccination activists (Jacobson et al., 2007), or by people who spread conspiracy theories (Lewandowsky et al., 2015, 2018). The underlying rationale of those inventories is that cognition that jettisons normative standards of truth-finding is unlikely to be a reality-tracking device, thereby affording a potential opportunity for people to learn to avoid such flawed argumentation. The present study shows that such learning can be achieved by a brief video.

Concerning the specific elements of inoculation in our training video (against hasty generalization, polarization and invoking emotion), it is worth noting that they have been implicated in arenas other than Islam-related radicalization. For example, Blassnig et al. (2019) showed that hasty generalizations are also commonly used by populist actors. Mooijman et al. (2018) found that moralizing language—which is often couched in emotional terms—was associated with indicators of violence during the 2015 Baltimore protests against police brutality. Similarly, heightened emotional states have been associated with increased endorsement of authoritarian policies (Vasilopoulos et al., 2018). A possible implication of those results is that our specific training video may also be effective in provided protection against misleading rhetoric in other domains. This intriguing possibility deserves to be explored in future research.

### Practical implications

Some interventions against violent extremism and radicalization have not relied on empirical evidence to inform best practice. For example, the US government program “Think Again, Turn Away” argued against Islamic State propaganda on social media. The program was unsuccessful and was eventually terminated. Critics argued that the program was beset with incompetence and lack of knowledge about the arguments it became involved in on Twitter (Katz, 2014). In the U.K., the government’s Prevent strategy, designed to stop people from becoming terrorists or supporting terrorism, has been subject to extensive, and sometimes withering, criticism (Awan, 2012; Qureshi, 2015; Richards, 2011; Thomas, 2010). Much of that criticism focused on the perceived stigmatization of the Muslim community.

These kinds of problems can be avoided in the inoculation framework because the material can be relatively generic. In the present study, the training video did not mention Islam or any issues related to Islam or radicalization. The video nonetheless successfully inoculated people against being misled by two diametrically opposed radicalizing positions. It follows that inoculation messages can be effective without the problems that beset other programs: neither lack of domain knowledge nor stigmatization are likely to derail inoculation.

Future research should test the effectiveness of inoculation on groups who are likely targets of extremists. Whereas our approach was generic and broad based, this may be insufficient to reach and protect at-risk populations. For example, adolescents are among the most active consumers of social media, which increases the risk of exposure to propaganda (Baugut & Neumann, 2019). An investigation in Germany reported that more than one third (37%) of participants aged 14–19 years had been exposed to radical content (Nienierza et al., 2019), and in the US, 57% of 320 surveyed students aged 14–19 said they had encountered hate speech on social media (Harriman et al., 2020). In the UK, a review of UK Government figures, civil society reports, reports from social media platforms, measurement studies, academic reports and survey data found that 41% of 18–30-year-olds had encountered cruel or hateful content online, compared with only 7% of older (76+) adults (Vidgen et al., 2019). It is particularly concerning that young people constitute the largest share of consumers of YouTube (Gottfried & Shearer, 2016), because young people may be unaware of the nature of the recommender system (Schmitt et al., 2018), and therefore may be more susceptible to its radicalizing influence than adults. Inoculation could give adolescents the tools to identify extremist messages and subsequently increase their resistance to persuasive misinformation. However, to date, there has been surprisingly little inoculation research involving young people below college age. Roozenbeek and van der Linden (2018) conducted a preliminary study with high school students (age 16–19) which showed that playing a fake news game slightly increased students’ ability to detect fake news, but these results were suggestive only and are in need of replication.

Future work must also explore avenues to roll out inoculation at scale. However effective a treatment may be in the laboratory, it cannot make a difference in the real world unless users are exposed to it. One potential avenue would involve YouTube itself, ideally by linking the inoculation material into the recommender system such that it is recommended to people who are deemed likely to watch potentially radicalizing content. Although YouTube’s practices have been said to normalize racism on social media (Hokka, 2021), this seems to arise from a neoliberal interpretation of the “marketplace-of-ideas” notion (Hokka, 2021) rather than a deliberate effort to support or sustain racism. YouTube has a clear policy against hate speech (Google, n.d.) and has recently revised the recommender system to avoid problematic content including conspiracy theories (YouTube, 2019, 2020). The idea of using the recommender system to deploy public-service messages is therefore not far fetched. On the contrary, given that we live in “algorithmically infused societies who are shaped by deeply entangled algorithmic and human processes and behaviour” (Wagner et al., 2021, p. 197), harnessing such algorithms in the public interest should be of increasing interest to the social sciences. At a time when regulation of social media is increasingly being entertained by policy makers, in particular in the European Union (e.g. Lewandowsky et al., 2020), a large-scale rollout of inoculation would constitute a response—whether by regulation or self-initiative by the platforms—that does not incur the risk of censorship.

## Conclusions

Online radicalization driven by disinformation is of growing concern in many societies and has demonstrable adverse consequences offline. Interventions based on rebutting of misinformation face several challenges: First, the dynamically changing nature of radicalizing content makes timely deployment of specific countermessages difficult. Second, corrections are often only partially successful. Finally, people who have consumed disinformation are often difficult to reach with corrections.

We therefore explored another tool that can protect people against misleading rhetoric, namely argumentation-based inoculation. We successfully inoculated against both Islamophobic and Islamist disinformation using a video that was presented in a neutral context, thereby circumventing some of the problems associated with rebuttals. The results point to a scalable intervention against radicalization that can be deployed preemptively without requiring specific knowledge of radicalizing content.

## Supplementary Information

**Additional file 1.** YouTube analysis and verbatim scripts.

## Data Availability

The method and analysis plan were preregistered. The preregistration is available at https://osf.io/au9wh/. The data set with potentially identifying information removed and all analysis scripts and Markdown files are available at https://osf.io/4eh3x/. The files in the repository permit recompilation of this manuscript, including the analysis.
