# Supplementary material for: Inoculating against the spread of Islamophobic and radical-Islamist disinformation
Source: Cogn Res Princ Implic. 2021 Aug 19;6:57. doi: 10.1186/s41235-021-00323-z (PMC8374109; doi:10.1186/s41235-021-00323-z)
Supplement: Supplementary file 1 — Additional file 1. YouTube analysis and verbatim scripts. [file 41235_2021_323_MOESM1_ESM.pdf]

Online Supplementary Material for: Inoculating Against the Spread of Islamophobic and Radical-Islamist Disinformation

Stephan Lewandowsky & Muhsin Yesilada

Online Supplementary Material for: Inoculating Against the Spread of Islamophobic and Radical-Islamist Disinformation

Section S1: Examining the YouTube landscape in relation to Islam-related Extremism

We aimed to explore the recommender system on YouTube to illustrate representative paths that users might take to make contact with extremist disinformation. The analysis considered the role of benign, apolitical, and non-violent search terms, and how they can lead to extremist content and disinformation after a few clicks. The study also explored paths to radical content from search terms that are likely to elicit anti-Islam and pro-Jihadi propaganda content, respectively.

Data collection

We identified four search keys to build Islamophobic and radical-Islamist networks, respectively (see Table S1 for the search keys). The analysis selected two overtly pro-jihadi and Islamophobic search items (identified with a double asterisk in the table) and two benign, apolitical search keys (single asterisk).

The search keys associated with radical-Islamist content were derived from a study that analysed the google search queries that returned problematic content (Ahmed & George, 2017). The search keys associated with Islamophobic content were derived from text analysis of Islamophobia on Twitter (Evolvi, 2018).

The online tool YTDT Video Network was used to build a list of related videos—that is, those recommended by the recommender system—from the search keys (Rieder, 2015). The tool retrieved related videos for each search key together with their metadata (e.g., video ID, video title, URL) from YouTube's application programming interface (API). Resources are first sorted based on their relevance, then in reversed chronological order based on the date they were created, their rating (highest to lowest), title (alphabetically) and view

count (highest to lowest number of view counts).

YTDT provides information about the related videos in the form of nodes (videos) and edges (a connection via the recommender system between two nodes) The related videos were collected with a crawl depth = 1. Crawl depth specifies how far from the initial search key the script should go. Crawl depth = 0 retrieves the relations between the videos returned from the search keys. The iterations was sent to 1 which returns 50 videos for each search key at crawl depth = 0. Crawl depth = 1 determines the relation between the videos returned from each of the search key and their directly-related videos (videos recommended from the first set).

Procedure

The related videos derived from the search YTDT search were used to build two visual networks using the Gephi software (https://gephi.org/) with the "ForceAtlas2" algorithm, for Islamophobic and radical-Islamist content, respectively. A modularity analysis then identified distinct communities (i.e., clusters of highly related videos). Videos in the network (nodes) were assigned a betweenness centrality value. Betweenness centrality is a measure of how often a node appeared on the shortest path between two nodes, and thus is an indicator of the importance of that node to the network as a whole.

The top 30% of videos (ranked by betweenness centrality) in each community, were categorised by their content (e.g., blogging, gaming, Islamophobic content, radical Islamist content etc.) for both of the respective networks.

A content analysis was then conducted by the second author on the top 50 videos (ranked by betweenness centrality) that were categorised as Islamophobic. A parallel content analysis was conducted on the top 26 videos (ranked by betweenness centrality) that were categorised as potentially radical-Islamist. Only 26 videos were analysed in the radical-Islamist network because it contained only a small number of videos.

Based on previous work (e.g., Roozenbeek & Linden, 2019) augmented by our own conceptual analysis, the content analysis targeted the following techniques associated with the production of misinformation: polarisation, invoking emotions, spreading conspiracy theories, trolling people online, deflecting blame, and impersonating fake accounts, misrepresentations of scripture, and cherry-picked data. The analysis also aimed to identify examples of common right-wing populist fallacies, based on prior work by Blassnig, Büchel, Ernst, and Engesser (2019): Ad consequentiam, Ad hominem, Ad populum, and hasty generalisations.

Results

Network structure. Figure S1 shows the networks for Islamophobia (on the left) and Islamism (right). The Islamophobia network comprised 8,972 nodes (videos) and 211,410 edges. The total number of communities in this network was 21, with a modularity value of 0.550, indicating medium to high distinctiveness between the communities. Communities 0, 10, and 6 comprised a large proportion of the nodes in the network; see legend in Figure S1 for a summary of the top 8 community sizes. Descriptive labels for the communities (based on content analysis) are shown in Table S2. The Islamist network comprised 11,367 nodes and 211,410 edges with 32 communities, with a modularity value of 0.610, indicating high distinctiveness between the communities. Communities 7, 29, and 28 comprised a large proportion of the nodes in the network (see legend). Descriptive labels for the communities are shown in Table S3.

Content analysis. For Islamophia, a content analysis of the top 30% of videos in each community, as measured by betweenness centrality, indicated that 13.45% of videos contained Islamophobic content. Communities 0, 10, and 6 contained the largest proportions of Islamophobic content; see Table S2 for a summary. For Islamist videos, the content analysis of the top 30% of videos in each community, as measured by betweenness centrality, indicated that 0.93% of the videos contained radical-Islamist content. Communities 3, 5, 6,

10 contained the largest proportions of radical-Islamist content in the network (Table S3).

Misinformation techniques. Figure S2 compares the number of occurrences of the specific misinformation techniques targeted by the content analysis between the two types of videos. The numbers plotted in the figure refer to the average number of occurrences of a technique per video of each class.

Some key differences between the networks are apparent: The Islamist videos exhibited more frequent use of misrepresentation of scripture, invoking emotion, and spreading conspiracies in comparison to the Islamophobic network. The Islamophobic network exhibited more frequent use of cherry-picked data in comparison to the Islamist network.

Section S2: Islamophobic and radical-Islamist scripts of the target videos

The scripts were designed to use the misinformation techniques being explained in the training video. The color coding refers to the three techniques Polarisation; Hasty Generalisations; and Invoking Emotion.

Islamophobic Content	Radical Islamist Content
**Islam in Society – A change is needed**	**Islam in society - A change is needed**
There is a definite problem with radical Islam in this country; altering our western democratic way of life. I mean whether we like it or not, and we can be very politically correct, but whether we like it or not, there is a situation. Islamic values are not in line with western values. To come up with a resolution; we have to be to be able to talk about the problem, or at least acknowledge that Islamic culture does not accommodate western culture. The recent events that have transpired have made it clear that we are under attack by Islam. This is clearly important.<br><br>The current state of Islam is transforming the western way of life. Parents have to change their behaviours as a means of	There is a definite problem with the westernisation in this country; altering our Islamic spiritual way of life. I mean whether we like it or not, and we can be very politically correct, but whether we like it or not, there is a situation. Westernisation is not in line with Islamic values. To come up with a resolution; we have to be to be able to talk about the problem, or at least acknowledge that westernisation does not accommodate Islamic culture. The recent events that have transpired have made it clear that we are under attack by westernization. This is clearly important.<br><br>The current state of westernization is transforming the Islamic way of life. Muslim parents have to change their behaviours as

91

raising their children up traditionally, without Islamic influence. This is one of the greatest things that change can offer us; an education system, media, and an environment that supports western values. The differences between the two groups are obvious and divide is needed.	a means of raising their children up Islamically, without westernization. This is one of the greatest things that change can of us; education system, media, and an environment that supports Islamic values. The differences between the two groups are obvious and divide is needed.
The wealth imbalance, the mass poverty that is spread all over the country; is a product of accommodating Muslims at the expense of western values. We have all heard about the decline in living standards, and housing across the country; and Muslim migration is at the root of the difference. It is clear that without Muslim migration, this mass poverty could be resolved.	The wealth imbalance, the mass poverty that is spread all over the country; is a product of accommodating westernization at the expense of Islam. We have all heard about the decline in living standards, and housing across the country; and capitalism is at the root of the difference. It is clear that without westernization, this mass poverty could be resolved.
For too long people have been deprived freedom of speech and the protests against Muslims have made it very clear that Islam is not welcomed in this country. Therefore, it is reasonable to propose the idea of moving away from the influence of Islam in	For too long Muslims have been deprived freedom of speech and the protests against Muslims have made it very clear that Islam is not welcomed in this country. Therefore, it is reasonable to propose the idea of moving away from the influence of

this country. It is reasonable to suggest that such an idea will bring back our freedom and prosperity. Islam is not working for its people and only westernization can protect the people. The country has been through a challenging time and it has happened at the same time as the rise of Islam. Therefore, only western values can put us back on track.	westernization in this country. It is reasonable to suggest that such an idea will bring back our freedom and prosperity. Westernization is not working for its people and only Islam can protect the people. The country has been through a challenging time and it has happened at the same time as the rise of westernization. Therefore, only Islamic values can put us back on track.
Take the recent events as a prime example. Fifteen people were wounded in a recent radical Islamic attack that has changed the lives of the individuals forever. Muslims who knew the perpetrator did not report the attack. If they did, they could have stopped the situation from occurring. It is now a reality for the victims; they will suffer because of a lack of action taken by the Islamic community. So it begs the question, how many examples do we put up with before we look for a solution?	Take the recent events as a prime example. Fifteen Muslims were wounded in a recent far-right extremist attack that has changed the lives of the individuals forever. Supporters who knew the perpetrator did not report the attack. If they did, they could have stopped the situation from occurring. It is now a reality for the victims; they will suffer because of a lack of action taken by the western community. So it begs the question, how many examples do we put up with before we look for a solution?

93

This situation is global; these situations are happening around the world at a high rate, and consistently. Look at the global incidences being reported, and you will see that Muslims do not report. How many times have you preventable scenes due to Muslims not acting on the situations? Many countries have identified that Muslims do not care about western values. And this fact could explain why there have been so many unreported situations, resulting in so many preventable events. We urge you to consider whether you are willing to accept these differences, or do you also crave direct action? Your opinion is very important, speak up.	This situation is global; these situations are happening around the world at a high rate, and consistently. Look at the global incidences being reported, and you will see that politicians do not report. How many times have you witnessed preventable scenes due to westerners not acting on the situations? Many countries have identified that westerners do not care about Islamic values. And this fact could explain why there have been so many unreported situations, resulting in so many preventable events. We urge you to consider whether you are willing to accept these differences, or do you also crave direct action? Your opinion is very important, speak up.

References

Ahmed, M., & George, F. L. (2017). A war of keywords: How extremists are exploiting the internet and what to do about it. *Center on Religion and Geopolitics.*

Blassnig, S., Büchel, F., Ernst, N., & Engesser, S. (2019). Populism and informal fallacies: An analysis of right-wing populist rhetoric in election campaigns. *Argumentation*, *33*, 107–136. doi:10.1007/s10503-018-9461-2

Evolvi, G. (2018). Hate in a tweet: Exploring internet-based islamophobic discourses. *Religions*, *9*, 307. doi:10.3390/rel9100307

Rieder, B. (2015). *YTDT video network.* Retrieved from https://tools.digitalmethods.net/netvizz/youtube/mod_videos_net.php

Roozenbeek, J., & Linden, S. van der. (2019). Fake news game confers psychological resistance against online misinformation. *Palgrave Communications*, *5*. doi:10.1057/s41599-019-0279-9

Table S1

Search Items and videos. See text for explanation of asterisks.

Risk of Radical-Islamist Content	Risk of Islamophobic Content
Caliphate *	Islam is the problem **
Killing infidels **	Muslim Migration *
No life without Jihad**	Islamic Grooming Gangs **
Sharia Law*	Islam United Kingdom *

Table S2

Summary of Islamophobia network

Group	Group label	N total	N top 30%	N top 30% Islamo-phobic	% in top 30% Islamo-phobic
C0	IC – Migration Concerns	1604	481	45	9.4
C1	Documentaries	659	198	5	2.53
C2	News & Politics	833	250	9	3.6
C3	IC - Grooming Gangs	521	156	25	16
C4	IC – Migration Concerns	195	59	6	9.74
C5	IC – Violence Concerns	773	232	89	38.36
C6	IC – Grooming Gangs	1209	363	71	19.56
C7	IC – Migration Concerns	319	96	18	18.75
C8	-	-	-	-	-
C9	-	-	-	-	-
C10	IC – Diverse Content	1366	410	72	17.6
C11	Religion & Spirituality	93	28	1	3.7
C12	News & Politics	249	75	5	6.66
C13	Sports	57	17	0	0
C14	Finance	52	16	0	0
C15	Entertainment	107	32	0	0
C16	-	-	-	-	-
C17	Education	41	12	0	0
C18	Religion & Spirituality	85	26	0	0
C19	Entertainment	183	55	0	0

C20	People & Blogs	130	39	0	0

Table S3

Summary of Islamophobia network

Group	Group label	N total	N top 30%	N top 30% Islamo-phobic	% in top 30% Islamo-phobic
C0	News & Politics	867	260	4	1.53
C1	Propaganda	680	204	8	3.92
C2	Propaganda	221	66	3	4.54
C3	Entertainment	92	28	0	0
C4	-	-	-	-	-
C5	Religion & Spirituality	77	23	0	0
C6	Religion & Spirituality	47	14	0	0
C7	Islamic State News videos	1774	532	13	2.44
C8	Education	36	11	0	0
C9	Gaming	72	22	0	0
C10	Gaming	87	26		0
C11	Islamophobic Content	70	21	1	4.59
C12	Movie Trailers	137	41	0	0
C13	People & Blogs	286	86	0	0
C14	Entertainment	831	249	0	0
C15	Entertainment	81	24	0	0
C16	Documentaries	88	24	0	0
C17	Music	240	72	0	0
C18	Entertainment	133	40	0	0
C19	Religion & Spirituality	51	15	0	0

C20	People & Blogs	98	29	0	0
C21	Entertainment	85	29	0	0
C22	Music	120	36	0	0
C23	Religion & Spirituality	130	39	0	0
C24	Religion & Spirituality	214	65	0	0
C25	-	-	-	-	-
C26	Education	9	3	0	0
C27	Gaming	88	26	0	0
C28	Islamophobic Content	1487	446	26	5.82
C29	Islamophobic Content	1731	519	36	6.93
C30	Documentaries	131	45	0	0
C31	Education	24	7	0	0
C32	Religion & Spirituality	381	114	0	0

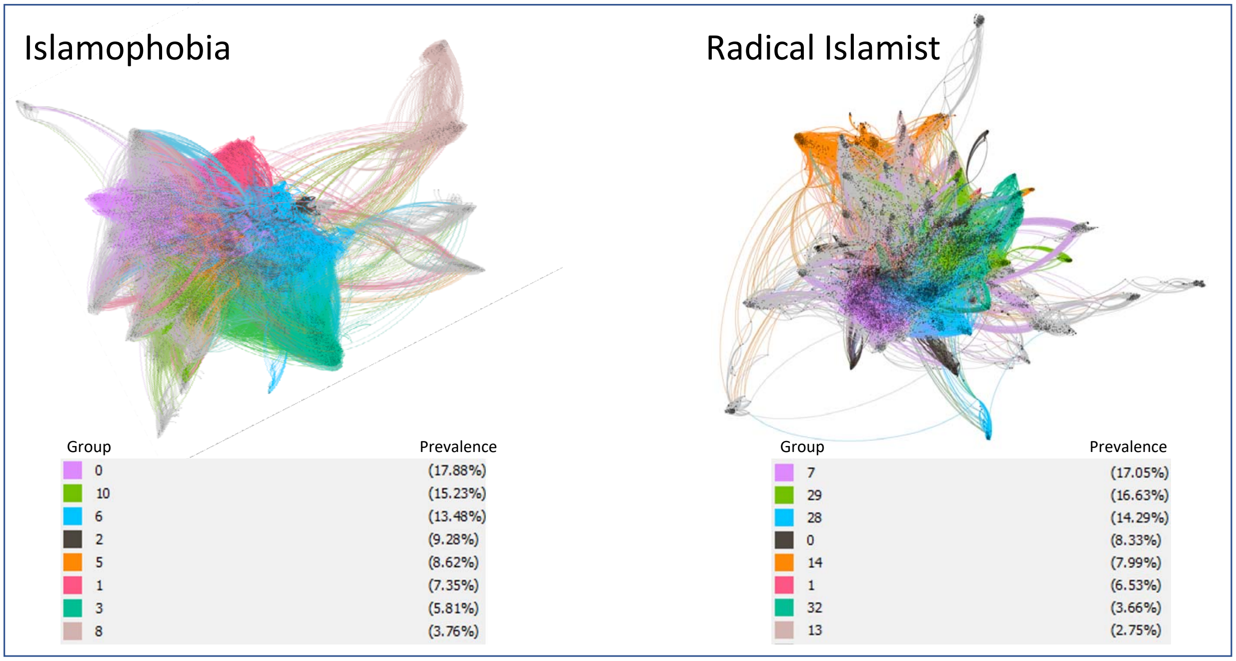

Figure S1. YouTube networks obtained with search keys targeting Islamophobic and Islamist content. Labels for groups are provided in Tables S3 and S2.

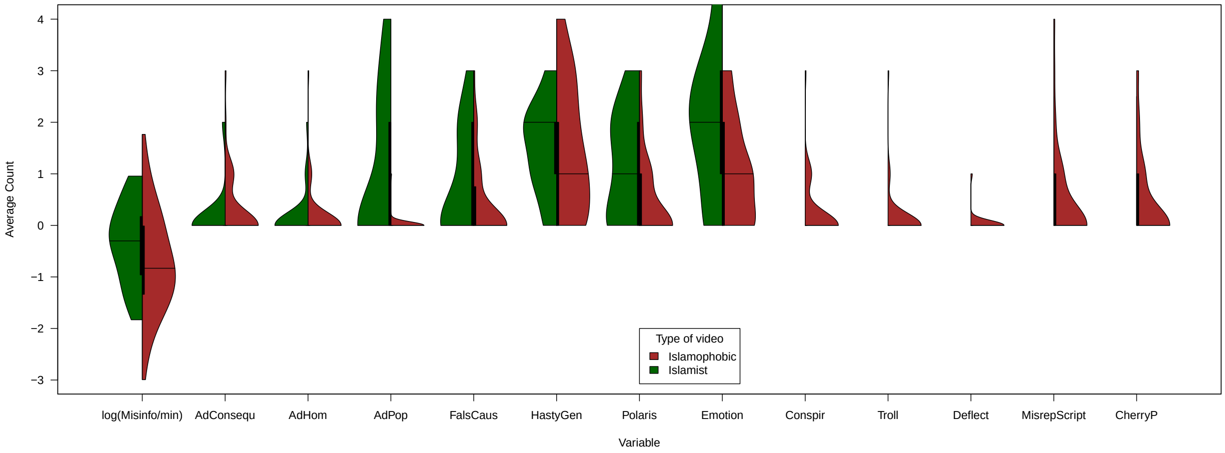

Figure S2. Average count per video of misinformation techniques identified in the Radical-Islamist (green) and Islamophobic (brown) networks. The misinformation count (number of misinformation occurrences per minute) is logarithmically transformed to permit plotting on the same scale. None of the other counts are transformed.
